# Evaluation of the Therapeutical Effect of Matricaria Chamomilla Extract vs. Galantamine on Animal Model Memory and Behavior Using 18F-FDG PET/MRI

**DOI:** 10.3390/cimb46050273

**Published:** 2024-05-09

**Authors:** Roxana Iacob, Matei Palimariciuc, Tudor Florea, Cosmin Vasilica Pricope, Cristina Mariana Uritu, Bogdan Ionel Tamba, Teodor Marian Ionescu, Cati Raluca Stolniceanu, Wael Jalloul, Romeo Petru Dobrin, Lucian Hritcu, Oana Cioanca, Monica Hancianu, Alexandru Gratian Naum, Cipriana Stefanescu

**Affiliations:** 1 Division of Nuclear Medicine, Department of Biophysics and Medical Physics, “Grigore T. Popa” University of Medicine and Pharmacy, 16 University Street, 700115 Iasi, Romania; 2 Department of Psychiatry, “Grigore T. Popa” University of Medicine and Pharmacy, 16 University Street, 700115 Iasi, Romania; 3 Advanced Center for Research and Development in Experimental Medicine (CEMEX), “Grigore T. Popa” University of Medicine and Pharmacy, 16 University Street, 700115 Iasi, Romania; 4 Department of Biology, Faculty of Biology, Alexandru Ioan Cuza University of Iasi, 700506 Iasi, Romania; 5 Department of Pharmacognosy, Faculty of Pharmacy, “Grigore T. Popa” University of Medicine and Pharmacy, 16 University Street, 700115 Iasi, Romania

**Keywords:** Matricaria chamomilla extract, enriched formula, scopolamine induced amnesia, cerebral metabolic profile, 18F-FDG PET, Alzheimer’s disease

## Abstract

The memory-enhancing activity of Matricaria chamomilla hydroalcoholic extract (MCE) is already being investigated by behavioral and biochemical assays in scopolamine-induced amnesia rat models, while the effects of scopolamine (Sco) on cerebral glucose metabolism are examined as well. Nevertheless, the study of the metabolic profile determined by an enriched MCE has not been performed before. The present experiments compared metabolic quantification in characteristic cerebral regions and behavioral characteristics for normal, only diseased, diseased, and MCE- vs. Galantamine (Gal)-treated Wistar rats. A memory deficit was induced by four weeks of daily intraperitoneal Sco injection. Starting on the eighth day, the treatment was intraperitoneally administered 30 min after Sco injection for a period of three weeks. The memory assessment comprised three maze tests. Glucose metabolism was quantified after the 18F-FDG PET examination. The right amygdala, piriform, and entorhinal cortex showed the highest differential radiopharmaceutical uptake of the 50 regions analyzed. Rats treated with MCE show metabolic similarity with normal rats, while the Gal-treated group shows features closer to the diseased group. Behavioral assessments evidenced a less anxious status and a better locomotor activity manifested by the MCE-treated group compared to the Gal-treated group. These findings prove evident metabolic ameliorative qualities of MCE over Gal classic treatment, suggesting that the extract could be a potent neuropharmacological agent against amnesia.

## 1. Introduction

Alzheimer’s disease (AD) describes 60–70% of cases affecting more than 55 million people worldwide, a debilitating disease whose incidence could potentially triple in the next 30 years [1].

The adult process of neurogenesis is altered in the earliest stages of Alzheimer’s disease (AD), by progressive and irreversible loss of memory, of higher cerebral functions and later, of the self-help skill [2]. Structurally, the hippocampal areas show some rates of atrophy in the earliest stages of the disease, while the precuneus and posterior cingulate show consistent and increasing rates of atrophy later. These are useful indices of progression of AD [3,4].

On the other hand, the brain has the highest avidity for glucose, consuming about 20% of the energy derived from its metabolism [5]. But in AD, glucose metabolism is dramatically decreased, partly due to oxidative damage to enzymes involved in glycolysis, in the tricarboxylic acid cycle and in adenosine triphosphate (ATP) biosynthesis, resulting in synaptic dysfunction and neuronal death and thus the thinning of important areas of the cerebral cortex [6,7,8]. The most involved hypometabolic cerebral regions are the hippocampus, posterior cingulate, precuneal, retrosplenial and limbic cortices [9,10,11].

Therefore, changes brought about by the onset of dementia can be best assessed by a combination of biomarkers. The comprehensive biochemical arsenal includes serum oxidation markers (e.g., cerebral acetylcholinesterase, superoxide dismutase, malondialdehyde, and so on) and cerebrospinal fluid markers (e.g., beta amyloid fractions, hyperphosphorylated tau proteins, and Apolipoprotein-E genotype) [12]. The imaging attributes entail positron emission tomography with a glucose analog radiopharmaceutical such as fluor-18 fluorodeoxyglucose (18F-FDG PET), as well as structural and functional magnetic resonance imaging (MRI), combined with machine-learning techniques. 18F-FDG PET examination alone can detect prodromal AD and highlight the progressive conversion of amnestic mild cognitive impairment (aMCI) to AD with an accuracy of 89% in a follow-up time of 5 years at the minimum [13,14], but in combination, all these complementary biomarkers can predict aMCI conversion to irreversible AD with an accuracy of almost 94% [15,16].

At the same time, the effectiveness of a long-term classic treatment of AD, represented by alkaloids with acetylcholinesterase-inhibiting proprieties (such as donepezil, galantamine, and rivastigmine), induces a significant metabolic increase in the specific brain areas [17] but does not determine a definitive cure of the disease.

Also, it is already known that Scopolamine (Sco) induces cognitive impairment in experimental animals by a muscarinic acetylcholine receptor blockade and determines an increase in the regional cerebral metabolic rate of glucose (rCMRglu) in all the cerebral regions of interest except the cerebellum and thalamus. These effects disappear with the co-administration of acetylcholinesterase inhibitors and Sco [18].

That is why there are all kinds of attempts to identify better treatments for the disease, including the Cioanca O. preclinical studies of antioxidant molecules that enter the brain and exert protective properties. As such, *Matricaria chamomilla* L. is a medicinal herb traditionally known to have anti-inflammatory, antimicrobial, antiviral, anxiolytic, and antidepressant proprieties. Supporting evidence demonstrated neuroprotective roles, memory-enhancing activity, and antioxidant properties roles [19,20]. Chamomile flowers are generally recognized as sources of flavonoids with good anti-inflammatory and antioxidant activity. Among the most common compounds in this medicinal plant, luteolin and apigenin derivatives are better represented. Recent studies indicated that luteolin has a protective effect against inflammation and increased glucose levels [21]. The hydroalcoholic extract of Matricaria chamomilla L (MCE) could be a potent neuropharmacological agent against amnesia via modulating cholinergic activity, neuroinflammation, and promoting antioxidant action in the rat hippocampus [22]. In AD treatments, because of the ease of administration, these hydroalcoholic extract could represent a definite area of research in the future. But functional imaging data to support these promising results still does not exist.

Starting from these premises, our goal is to find quantifiable 18F-FDG PET functional imaging data from the study of AD Sco-induced animal models to highlight the metabolic changes that support the therapeutic proprieties of MCE.

## 2. Materials and Methods

### 2.1. Plant Extract Presentation

Dried chamomile flowers were used to obtain an alcoholic extract in ratio of 1:10 plant material in ethanol (70%). The raw material (bags of 40 g) was harvested in the summer and purchased in the fall of 2020, from a local producer (coordinates 45°49′55.8″ N 23°12′21.3″ E, Fares SA, Orastie, Romania). The identified flowers were given a code number (MC 38.01.21) for preservation purposes and for further reference, they were included in the sample herbarium from the Department of Pharmacognosy and Phytotherapy of the Faculty of Pharmacy, “Grigore T. Popa” University of Medicine and Pharmacy, Iasi, Romania.

The extraction procedure was like the one described by Ionita et al., 2018 [22], with some modifications. Briefly, 10 g of plant material was sequentially extracted on a thermostatic water bath at 85 °C, in a final volume of 100 mL of 70% ethanol. Each extraction used 45 mL of solvent. The obtained solutions were pooled together, and the last amount of 10 mL was used to wash the flask and then filter to reach a final volume of 100 mL in a volumetric flask. The obtained solution was then concentrated in a rotary evaporator to a total volume of 5 mL.

Given the fact that the chemical composition of a Matricaria chamomile flower extract is generally known, we improved the final extract by adding 1 mg of luteolin for each mL of fluid extract. Then, the extract was concentrated until dryness and kept in a tight air container, away from light and heat (fridge, 4–8 °C).

The chemical profile of the obtained extract was evaluated by comparing the UHPLC fingerprint to the previous profile of a standardized chamomile extract at 280 nm.

Prior to its use, the extract was dissolved in saline for intraperitoneal administration.

### 2.2. Animal Experiments

The ethical permit number 38/07.02.2021 was assigned to the protocols of our prospective interventional study by the Committee on the Ethics of Animal Experiments of the “Grigore T. Popa” University of Medicine and Pharmacy, Iasi, Romania. Also, the authorization number 37/09.07.2021 was issued by the Veterinary Sanitary and Food Safety Directorates, Iasi, Romania. The procedures were accomplished in accordance with the Directive (2010/63/EU, 22.09.2010) of the European Parliament and the Council of the European Union on the protection of laboratory animals. We used the minimal number of animals, and we lessened as possible their suffering.

Adult male Wistar rats (two months old), weighting 220 ± 25 g, were purchased from “Cantacuzino” Institute (Bucharest, Romania) and were maintained under temperature, humidity, and light-controlled conditions (24 ± 2 °C, 50 ± 5%, a 12 h light/dark cycle starting at 07:00 h) with tap water ad libitum and food half of their ad libitum intake. The animal species and breed were chosen following the indications of Mazen Asaad and Jin Hyung Lee in their guide to study Alzheimer’s disease in animal models [23].

The rats were divided into four groups, including the following:

- the Negative Control group (NC) received 0.2 mL physiological saline, intraperitoneally, for the first 28 days of the experiment;
- the Positive Control group (PC) or the Scopolamine-induced group received intraperitoneally, for 28 consecutive days, 2 mg/kg bodyweight Sco, an anticholinergic drug, dissolved in 0.2 mL physiological saline;
- the Classic Treatment group (Sco + Gal), that from the 8th day of the study and during 21 consecutive days, received intraperitoneally the specific treatment for AD, represented by 3 mg/kg bodyweight Galantamine (Gal), an alkaloid with acetylcholinesterase-inhibiting properties, 30 min after Sco administration;
- the Matricaria Chamomilla Extract Treatment group (Sco + MCE), from the 8th day of the study and during 21 consecutive days, received 75 mg/kg bodyweight of MCE intraperitoneally, 30 min after Sco administration.

The solutions were injected in the same volume for all groups, represented by 0.2 mL per intraperitoneal administration. To exclude diurnal variations of brain metabolism, all the experiments were performed between 8:00 AM and 2:00 PM.

### 2.3. Blood Glucose Level and Bodyweight Assessment

Blood glucose was collected from the tail veins of each rat and measured by a blood glucometer, à jeun, 30–60 min before image acquisition, one time for each rat, at the 16th or 23rd day of the experiment. Body weight assessment was performed once every week for the first five weeks and on the last day of the experiment.

### 2.4. Brain Image Acquisition and Processing

#### 2.4.1. Animal Preparation

The rats received no meal on the morning of the PET assessment (no more than 12 h fast time). For each animal, 18F-FDG (commercial vendor: Monrol Europe S.R.L, Bucuresti, Romania), 32.04 ± 6.3 MBq in 0.2 mL saline, was injected in a lateral tail vein. The animal was then kept in a dark room. After 45 min, the animal was placed in a plexiglass chamber and was anesthetized using an isoflurane delivery system at an induction concentration of 4% and a maintenance concentration of 2% isoflurane in a mixture of air and oxygen (no more than 1 h anesthesia time).

#### 2.4.2. 18F-FDG Brain PET/MRI—Image Acquisition

The 18F-FDG PET scans were obtained using an animal-dedicated PET/MRI scanner (nanoScan, Mediso Ltd., Budapest, Hungary) which was installed in the Advanced Center for Research and Development in Experimental Medicine (CEMEX) of “Grigore T. Popa” University of Medicine and Pharmacy, Iasi, Romania. Technical characteristics of this scanner were described before [24,25]. 3D PET images were reconstructed using a Maximum Likelihood Estimation Method (MLEM) algorithm (8 iterations and 3 subsets), with a slice thickness of 3 mm. The acquisitions were centered on the head region of each rat and were accomplished in the third week of the experiment. Image acquisitions were performed in the prone position, starting from 30 min after FDG administration with the MRI localization scan, and subsequently continuing with the PET scan 45 min after the radiotracer administration.

MRI data acquisition comprised three series of images as follows: a Fast Scout Sagittal Series, a GRE 3D Multi FOV Series, and a multi-echo 2D fast spin echo sequence (T2 FSE 2D) Axial Series, with very long TR: 8162.9 ms, normal TE: 55.1 ms, FA: 30°, NSA: 4, and slice thickness: 1.2 mm. These images provide a good contrast between gray and white matter, and between the brain and other soft tissues, and only need a short investigation time of 12 min for the axial plane.

PET data acquisition comprised an image series, including a static acquisition of 30 min Tera-Tomo 3D Full Model, with a MLEM of image reconstruction (8 iterations and 3 subsets), 400–600 keV, 1:5, slice thickness: 3 mm.

#### 2.4.3. Image Processing

**Figure 1:**
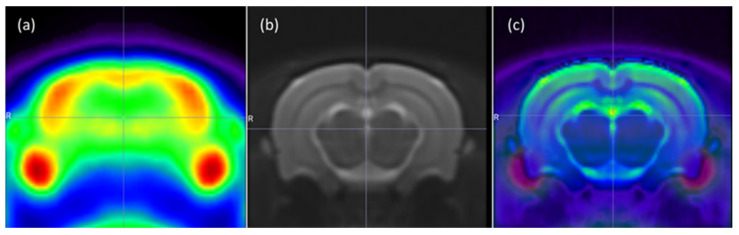
Coronal section of: (**a**) 18F-FDG PET brain template, on a Rainbow Color Scale; (**b**) reference MRI template, on a Gray Color Scale; and (**c**) fused 18F-FDG PET/MRI. Letter “R” represents the right side of the image.

The reconstructed PET DICOM images were imported into Statistical Parametric Mapping software (SPM12; Revision Number 7771; http://www.fil.ion.ucl.ac.uk/spm/, accessed on 5 April 2022; Wellcome Trust Centre for Neuroimaging, University College London, London, UK). First, images were converted to NIfTI format, and then the Small Animal Imaging Toolbox (SAMIT v1.3; Revision Number 1.3.1; http://mic-umcg.github.io/samit/, accessed on 5 April 2022; University of Groningen, University Medical Center Groningen, Medical Imaging Center, Groningen, The Netherlands) was employed for examining differences in the brain activity. Briefly, a tracer-specific template was built [26,27] by spatial normalization, co-registration of the images to a reference MRI template and brain masking of PET images which separates the brain from surrounding tissue. An example of these templates can be seen in Figure 1.

**Figure 2:**
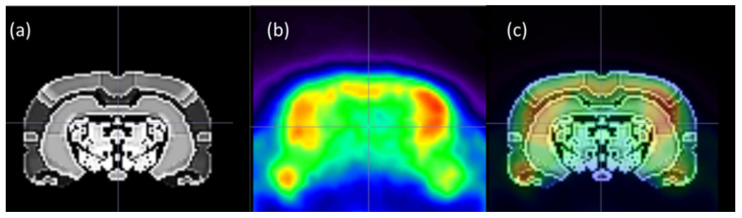
Coronal section of the (**a**) Schwartz atlas VOI template, on a Gray Color Scale; (**b**) 18F-FDG PET Wistar brain rat, on a Rainbow Color Scale; and (**c**) fused images highlight the automated VOI for SUVglc parametric images analysis.

The integrated Schwartz brain atlas was used for tissue segmentation of the brain data [28]. The volumes of interest (VOI) template atlas contains 50 symmetrical cortical and subcortical regions, as in Figure 2. To assess the brain glucose metabolism, 18F-FDG uptake was measured in different brain areas using the predefined VOIs for quantitative analysis, maximum and mean Standardized Uptake Values (SUVmax and SUVmean), and blood glucose-corrected Standardized Uptake Values (SUVglc) were automatically calculated. Quantification of the metabolic changes was made using known formulas for specific areas of the brain [29,30]. In all subjects, 18F-FDG uptake was visible in all cerebral areas and the cerebellum. Furthermore, the quantitative 18F-FDG accumulation expressed in the SUVmax, SUVmean, and SUVglc values was calculated for AD characteristic regions of the brain, including the whole brain, cingulate cortex, precuneus, caudate putamen, callosus cortex, entorhinal cortex, retrosplenial, globus pallidus, accumbens, amygdala, hippocampus, thalamus, and hypothalamus.

### 2.5. Behavioral Assays

The Y-maze tasks evaluate hippocampal lesions and medication effects on cognition by spontaneous behavior appreciation, and the spatial memory of rodents by the estimation of reward behavior. The Y-maze apparatus is made up of polyvinyl chloride material and consists of three identical arms (50 cm length × 10 cm width × 15 cm high walls) intersected at an angle of 120 degrees in the shape of a “Y”.

The Spontaneous Alternation Test (SAT), studied in the Y-maze, was conducted in the second week, after 3 days of treatment administration, as previously reported [31,32]. The rat is placed in the center of the maze and left to move freely for 5 min. If it chooses an arm other than the one explored just before, a correct answer is noted (alternation). The return to a previously explored arm is noted as a wrong answer (error). Each animal was tested for a 5 min session. The locomotor activity, implying the number of arm entries, and the percent spontaneous alternation, referring to total good entries divided by (total entries minus two), were analyzed. Good alternation means, e.g., ABC, ACB, BAC, CAB, and bad alternation means, e.g., ACA, BAB, CBC.

The Two Rewarded Alternation Tests (RAT) were conducted in the fifth and the sixth weeks of the experiment, after more than 17 days of treatment administration, for the first test, and respectively, 3 days after treatment cessation, for the second test. Each rat performed a number of 5 trials per test, one every 30 min. Initially, food rewards were placed in two arms of the maze, one of them being closed. The rat placed at the end of the third arm, explored the open arm and consumed a one-pellet reward. For the second entry, both arms were open, and the rat could choose the arm to explore. If the previously closed arm was chased, the rat could eat a new reward. The final score adds the number of rewards eaten and the second arm explorations for 5 trials with 2 entries. A perfect short memory, scoring 15 points, represents a complete task, while a poor memory, scoring 0 points, represents just one arm exploration and no food intake. This test was previously described by [33]. The rats were placed back into their home cages after each trial completion and olfactory clues were removed from the maze by alcohol wiping.

The elevated plus maze (EPM) task evaluates an animal’s anxiety-like behavior and locomotor activity. This apparatus is represented by four arms of polyvinyl chloride material, two open arms (a potentially stressful zone) cross-shaped with other two closed arms (a safe zone), 50 cm above the floor. The arms are 50 cm length × 10 cm width, while the two opposing arms have 15 cm high plastic walls [34,35].

In this model, the behavior assessment was conducted in the fifth week, 4 days after treatment cessation. The 8 min test started with the rat placed in the center of the maze, facing a closed arm. The registered behavioral parameters comprised the cumulative time spent in the open and in the closed arms (time expressed as a percentage) and the number of light/dark shifts. More time spent in the open arms means a less anxious behavior, and the total number of crosses represents the general locomotor activity of the rat [35,36,37].

A video camera recorded the animals’ behavior for all the tasks and the footage was later analyzed. Between the sessions, all the apparatus was cleaned using a 70% ethanol solution.

### 2.6. Statistical Analysis

All statistical analyses were performed using IBM SPSS Statistics for Windows, version 26 (IBM Corp., Armonk, NY, USA). All data were reported as mean ± standard deviation (SD). Differences between the means were considered statistically significant if *p* < 0.05.

## 3. Results

### 3.1. Chemical Composition of the Matricaria Chamomilla Extract (MCE)

The chemical profile of the MCE was similar to the standardized chamomile extract [22], and the major components identified for our extracts were apigenin, apigenin-7-O-glucoside, luteolin, luteolin-7-O-glucoside, rutoside, and chlorogenic acid. Table 1 indicates the quantified values for the investigated extract.

It is notable that the amount of luteolin is higher than apigenin and represents half of the apigenin-7-glucoside, but this is due to the enrichment of the MCE. Dependent on the environmental conditions, in chamomile extracts, the usual ratio of these three components would be 3:1:0.5 (apigenin-7-glucoside:apigenin:luteolin). However, for this research, we intended to increase the amount of luteolin. This flavonoid is recognized for its good anti-inflammatory potential and its mechanisms involve the inhibition of IL-6 and TNF-α synthesis in macrophages, leading to the improvement in glucose tolerance and insulin sensitivity in murine models [21]. The Supplementary Materials show the MCE chromatogram indicating the most important peaks and the standards at 330 nm in Figure S1. Also, the retention time for the used standards can be found in Table S1 and Figures S2–S10 represent UV spectrum for each component used standard: the Chlorogenic acid, the Cafeic acid, the Rutoside, the Hyperoside, the Luteolin-7-O-glucoside, the Apigenin-7-O-glucoside, the Quercetin, the Luteolin and the Apigenin.

### 3.2. Effects of Matricaria Chamomilla Extract on Brain Metabolism and Image Quantifications

The PC rats showed significantly higher Whole Brain SUVglc levels (2.49 ± 0.76 g/mL, range 0.63–8.09 g/mL) than the NC rats (1.96 ± 0.49 g/mL, range 0.61–5.38 g/mL, *t*-test: *p* < 0.001). The right amygdala showed the greatest increase (37% difference) in Sco-injected rats, while the right raphe nucleus showed the least (9% difference). The left (6% difference) and right (4% difference) pons showed a significant decrease in SUVglc levels.

**Figure 3:**
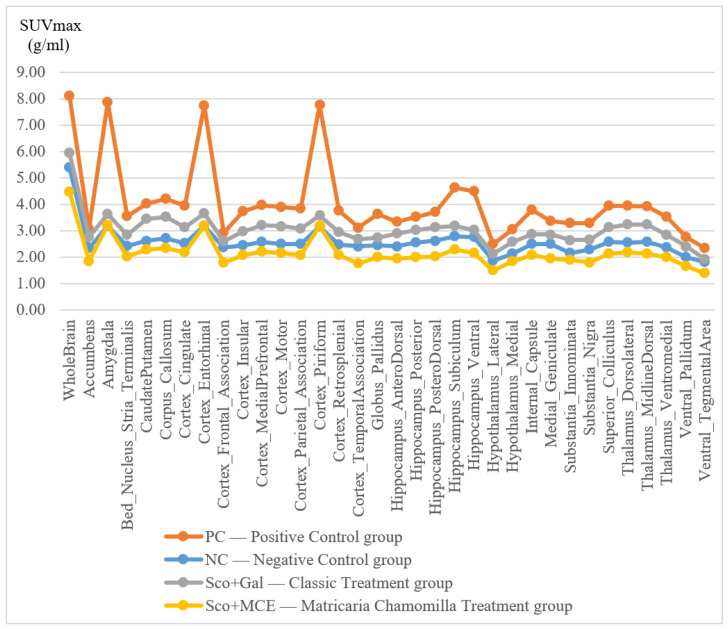
Metabolic effects of Matricaria chamomilla extract in AD specific cerebral regions, by 18F-FDG PET examination, after chronic treatment of Sco-induced cognitively impaired rats.

After 1 and 2 weeks of treatment, the Sco-induced rats treated with Matricaria chamomilla extract show metabolic similarity with normal rats, in the AD-specific cerebral regions, upon 18F-FDG PET examination. The Gal-treated group shows features closer to the PC group, as can be seen in Figure 3. We describe the following two examples: (1) the mean value of SUVmax in the cingulate cortex of the acetylcholinesterase-inhibitor-treated rats is 2.33 g/mL ± 0.16 SD, the PC rat has a SUVmax value of 2.53 g/mL, and the Matricaria-treated group presents 1.83 g/mL ± 0.16 SD, while NC has a 1.48 g/mL; (2) the mean value of SUVmax in the entorhinal cortex of Gal-treated rats is 2.7 g/mL ± 0.72 SD, the PC rat has a SUVmax value of 4.98 g/mL, and Matricaria-treated group presents 2.77 g/mL ± 0.72 SD, while NC has a 1.87 g/mL.

**Figure 4:**
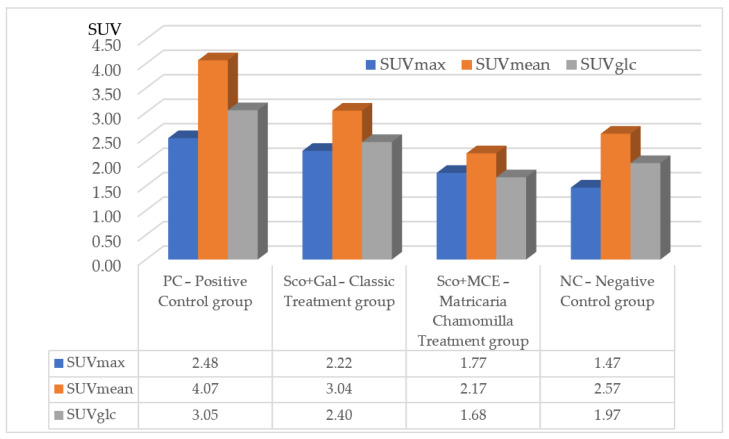
Comparison of metabolic features (SUVmax, SUVmean and SUVglc) of chronic Matricaria chamomilla extract treatment with Galantamine treatment, Positive and Negative Control groups, by 18F-FDG PET examination, in Sco-induced cognitively impaired rats.

These metabolic features are maintained regardless of the SUV calculation method (the maximum uptake, the mean uptake, or the glucose corrected formula) for glucose metabolism in all the characteristic cerebral regions. Sco-induced and Chamomilla-treated rats present SUV values closer to those of normal rats, as shown in Figure 4.

The right amygdala, piriform, and entorhinal cortex showed the highest differential 18F-FDG uptake (4.87, 4.78, and 4.68 g/mL) of the 50 regions analyzed.

### 3.3. Effects of Matricaria Chamomilla Extract on Behavioral Assessments

**Figure 5:**
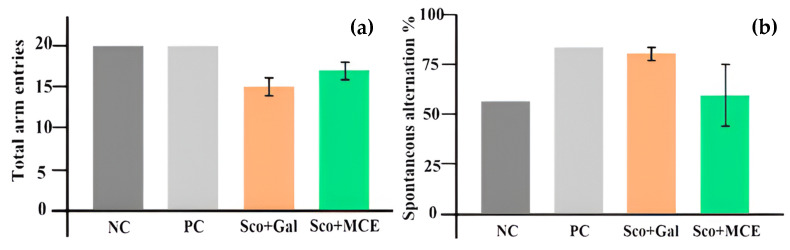
Effect of MCE as compared with the classic treatment (Gal), on the number of arm entries (**a**) and on the spontaneous alternation percentage (**b**) in the Y-maze SAT, in cognitive impairment Sco-induced rats.

Motor activity and spontaneous alternations were assessed in the Y-maze task, by the SAT. From the center of the maze, rats were allowed to move freely for 5 min. The NC group showed equal interest in all the arms (arm A, 6 entries; arm B, 7 entries; and arm C, 7 entries), with a total number of 20 entries and 56% spontaneous alternations. The Sco + Gal group had an average of 15 ± 1 total number of entries, with equal interest for all the arms (arm A, 5 entries; arm B, 5 entries; and arm C, 6 entries), and 79% spontaneous alternations. The Sco + MCE-treated group had an average of 17 ± 1 total number of entries, with an increased interest for the first arm (arm A, 7 ± 1 entries; arm B, 4.6 ± 1.5 entries; and arm C, 5.3 ± 0.5 entries), and 59% spontaneous alternations. The PC group showed equal interest in all the arms (arm A, 6 entries; arm B, 7 entries; and arm C, 7 entries), with a total number of 20 entries and 83% spontaneous alternations. A graphic comprising these results can be seen in Figure 5.

After 10 days of Sco administration and 3 days of treatment, the SAT makes no clear differentiation between the groups. The healthy, some of the ill and some of the treated rats manifested an increased percentage of correct spontaneous alternations and a large number of entries. The level of locomotor activity (represented by the total number of entries) is highest in the control group (20 entries), followed by the Sco + MCE-treated group (17 ± 1 entries). The animals showed a small alternation percentage for Sco + MCE group (59% ± 1.6%), closer to the normal group (56%). Sco + Gal group scored 79% ± 0.03%, closer to the PC group (83%).

**Figure 6:**
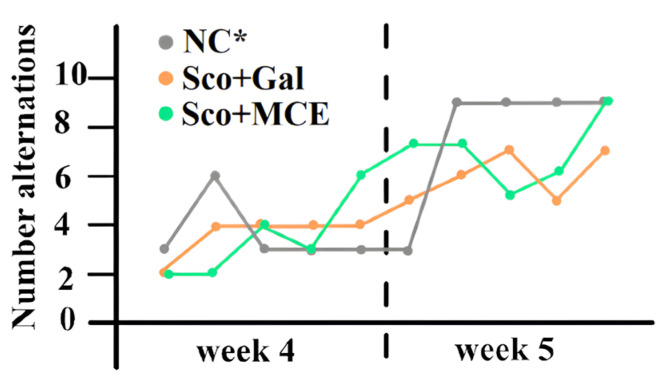
Effect of MCE, as compared with the classic treatment (Gal), on the spontaneous alternations in the Y-maze RAT in rats with Sco-induced cognitive impairment after 2 and 3 weeks of treatment. (*) alternation levels were adapted to the number of rats in the other groups.

Spatial working memory or short memory was evaluated in the Y-maze task by the RAT. The final score added the number of rewards eaten and the presence of second arm explorations for five trials with two entries. The NC group scored 6 points in the first test and 13 in the second. The Sco + Gal-treated group scored a mean of 6.3 ± 4 points in the first test and 10 ± 5 in the second test. The Sco + MCE-treated group scored a mean of 5.6 ± 2 points in the first test and 11.3 ± 4.7 in the second test. Both the classic-treated group and the MCE-treated group had few very agile rats that, in the second test, learned the task and received all the rewards, as well as few rats that did not accept the rewards in any trial. All groups showed an evolution from the first test, the best short term memory performance being obtained by the Sco + MCE-treated group in the second test, with new arm exploration in 86.6% of the cases, closer to the normal group score. The spontaneous alternations in the Y-maze RAT, after 2 and 3 weeks of treatment, are graphically presented in Figure 6.

**Figure 7:**
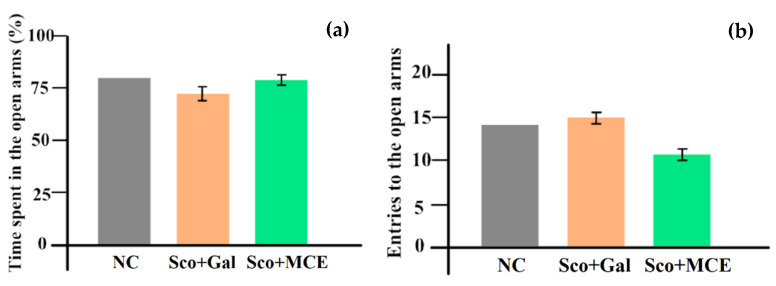
Effect of Matricaria chamomilla extract as compared with Galantamine, in rats with Sco-induced cognitive impairment. Percentage of time spent in the open arms (**a**) and number of entries to the open arms (**b**) in the elevated plus maze test.

The anxiety and locomotor activity were evaluated in the elevated plus maze (EPM) task (as presented in the Figure 7), concluding a less anxious behavior and a better activity manifested by the Sco + MCE-treated group compared to the Sco + Gal-treated group. So, this test highlighted that the NC spent 77.7% time in the open arms, with 27 crosses from dark to light and reverse. The acetylcholinesterase-inhibitor-treated group spent 71.7% ± 3.6% time in light, with 28.6 crosses and the Sco + MCE-treated group spent 77.5% ± 2.7% time in light, with 21 crosses.

## 4. Discussion

Matricaria chamomilla has been known as a traditional therapy since ancient times due to its anti-inflammatory, antioxidant, and disinfectant proprieties, with memory-enhancing, neuroprotective roles, and lack of toxicity for long-term administration. Previous studies of behavioral and molecular experiments demonstrated impaired memory processes in laboratory rats treated with scopolamine, effects ameliorated by MCE supplementary treatments [22]. These studies are also supported by our behavioral assessments. After 10 days of Sco-induction, including 3 days of Matricaria Chamomilla luteolin enriched treatment, the SAT makes no drastic differentiation between the groups, but the locomotor activity of MCE-treated group is almost as good as that of the normal control. Also, the MCE animals showed little willingness to explore new environments, similar to the normal rats. The Gal-treated group presented locomotor activity and percentage of spontaneous alternations closer to the diseased control. In the evaluation of short memory by the RAT, all groups showed an increase in the scores in the second test, as compared with the first test. The best short term memory performance was obtained by the MCE-treated group, very close to the normal rat score. The EPM test highlighted less anxious behavior and better locomotor activity manifested by the MCE-treated group compared to the acetylcholinesterase -inhibitor-treated group.

Then, it is known that a long-term treatment with acetylcholinesterase-inhibiting alkaloids induces a metabolic increase in AD-characteristic brain areas, but these effects disappear with the co-administration of Sco [18], so no definitive cure of AD is shown.

As far as we know, the assessment of cerebral tissue glucose metabolism after MCE treatment was not studied before. From the metabolic evaluation, we behold that Sco determines the elevation of cerebral glucose metabolism as compared with normal rats (*t*-test: *p* < 0.001). We assessed the usefulness of MCE chronic treatment. After 1 and 2 weeks of treatment, the metabolic features in the AD-specific cerebral regions of Sco-induced and MCE-treated rats grew close to those of normal rats, while the metabolism of the Gal-treated group increased and became close to the metabolic aspects of the affected group. The SUV calculation method for glucose metabolism (SUVmax, SUVmean or SUVglc) does not influence these metabolic features.

Thus, there is a correlation between the behavioral and metabolic assessments, with the Sco-induced and MCE-treated rats having metabolic features and maze performances similar to those of normal rats, while the Gal-treated group have features close to the Sco-induced and untreated rat.

Another novelty of this study is the enriched pharmaceutical formula of Matricaria Chamomilla hydroalcoholic extract that is evaluated by functional imagery, glucose metabolism, and behavioral parameters on an AD animal model. This research will allow for further translational studies of this natural extract on human patients because it has minimal to no side effects [38] thus increasing the compatibility of the treatment to patients.

The discussion of this study is not complete without some methodological considerations. We started with a small number of animals, that was further reduced by two unexpected, sudden deaths (rats of the positive control group). Without a doubt, a larger cohort of animals would exhibit more accurate values. But from previous studies [22], we know that Sco treatment determines the significant decrease in spontaneous alternation percentage and the increase in locomotor activity in contrast with the normal control group. Also, during the experiment, the PET/MRI scanner displayed a malfunction, which did not allow for a second image acquisition, to assess the dynamics of cerebral glucose metabolism. Carrying out this stage of the research is planned for a new study.

## 5. Conclusions

Despite all its shortcomings, our study proved better performances for the Matricaria-treated group compared to the Gal-treated group in all the behavioral evaluations (short-term memory, spontaneous and induced activity, and anxiety management), as well as the metabolic ameliorative qualities of MCE over the Gal classic treatment in AD-specific cerebral areas.

In addition to other studies on MCE showing the counteraction of oxidative stress and neuroinflammation for Scopolamine chronic diseased rats, the results of this research prove that our hydroalcoholic extract of Matricaria Chamomilla shows an improvement in memory impairment in behavioral tests, in correlation with the ameliorative effect on metabolic uptake in the AD-characteristic cerebral regions of interest. Having no adverse effects, MCE could be a potent neuropharmacological agent against amnesia of AD origin. This should be an impetus for further, larger investigations on the functional efficient neuroprotection of MCE to stop the extension of this devastating neurodegenerative disease.

## Figures and Tables

**Table 1:** The chemical fingerprint of the investigated MCE.

Sample	Concentration (µg/mL Extract)					
	Apigenin	Apigenin-7-Glucoside	Luteolin	Luteolin-7-Glucoside	Rutoside	Chlorogenic Acid
MCE	243.11	742.33	361.02	65.02	112.35	44.57

## Data Availability

Data are available upon request by mailing the corresponding author.

## Supplementary Materials

The following supporting information can be downloaded at: https://www.mdpi.com/article/10.3390/cimb46050273/s1, Figure S1: MCE chromatogram indicating the most important peaks and the reversed image for the standards at 330 nm. Table S1: Retention time for the used standards. Figure S2: UV spectrum for the Chlorogenic acid used standard. Figure S3: UV spectrum for the Cafeic acid used standard. Figure S4: UV spectrum for the Rutoside used standard. Figure S5: UV spectrum for the Hyperoside used standard. Figure S6: UV spectrum for the Luteolin-7-O-glucoside used standard. Figure S7: UV spectrum for the Apigenin-7-O-glucoside used standard. Figure S8: UV spectrum for the Quercetin used standard. Figure S9: UV spectrum for the Luteolin used standard. Figure S10: UV spectrum for the Apigenin used standard.
