# Supplementary material for: Evaluation of the Therapeutical Effect of Matricaria Chamomilla Extract vs. Galantamine on Animal Model Memory and Behavior Using 18F-FDG PET/MRI
Source: Curr Issues Mol Biol. 2024 May 9;46(5):4506–18. doi: 10.3390/cimb46050273 (PMC11119716; doi:10.3390/cimb46050273)
Supplement: Supplementary file 1 [file cimb-46-00273-s001.zip › cimb-2983256-supplementary.pdf]

Supplementary materials: Matricaria flower extract

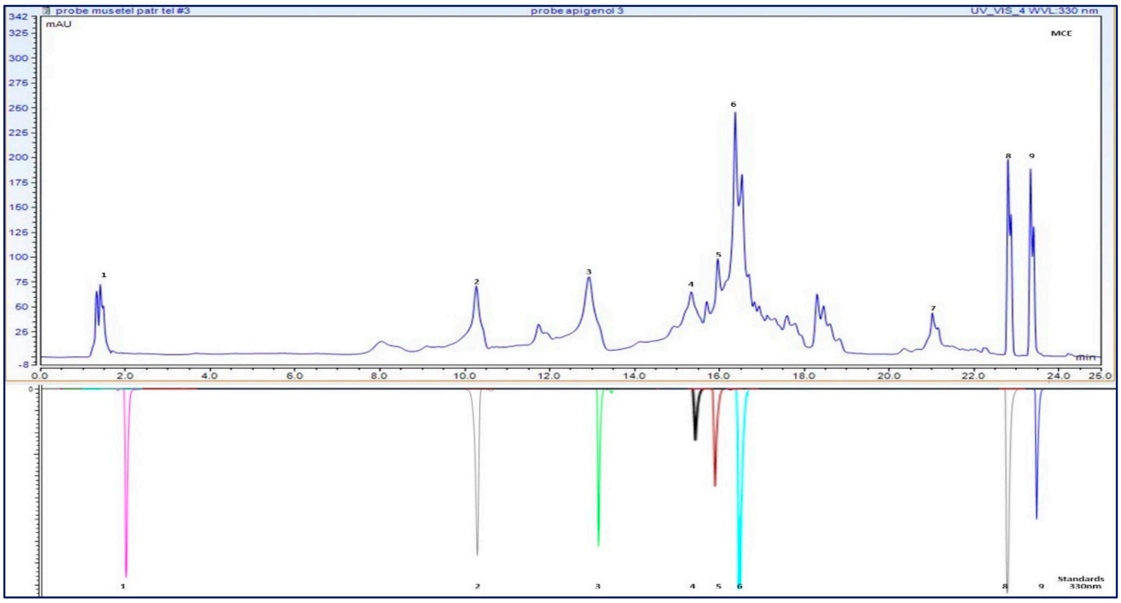

Supplementary Figure S1. MCE chromatogram indicating the most important peaks (the number of the compound is explained in the table below) and the reversed image for the standards at 330nm.

Supplementary Table S1. Retention time (Rt) for the used standards

Standard (no)	Rt (min)
Chlorogenic acid (1)	2.570
Caffeic acid (2)	10.217
Rutoside (3)	12.893
Hyperoside (4)	15.871
Luteolin-7-O-glucoside (5)	16.034
Apigenin-7-O-glucoside (6)	16.473
Quercetin (7)	21.123
Luteolin (8)	22.523
Apigenin (9)	23.397

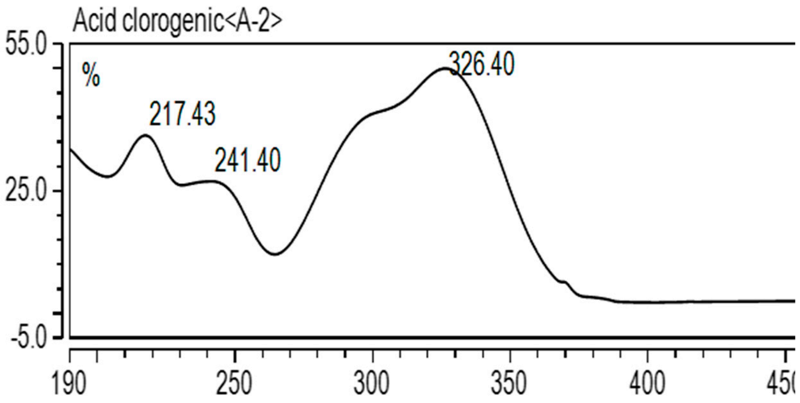

Supplementary Figure S2. UV spectrum for the Chlorogenic acid used standard

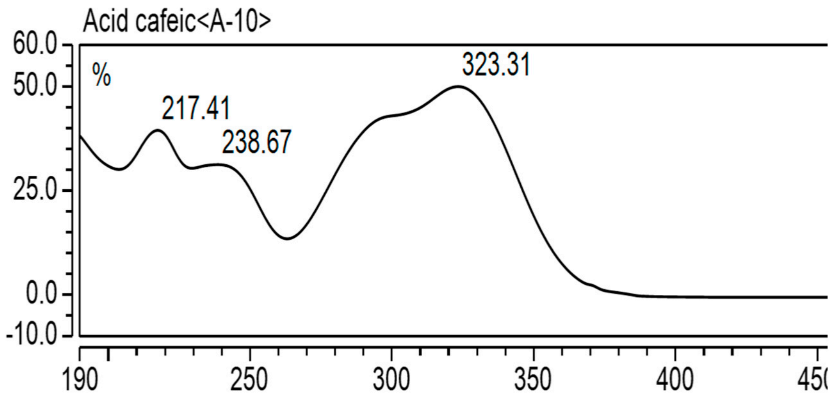

Supplementary Figure S3. UV spectrum for the Cafeic acid used standard

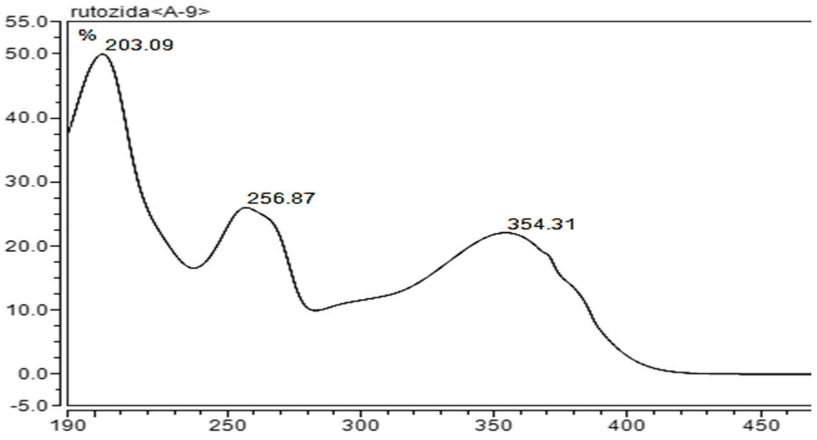

Supplementary Figure S4. UV spectrum for the Rutoside used standard

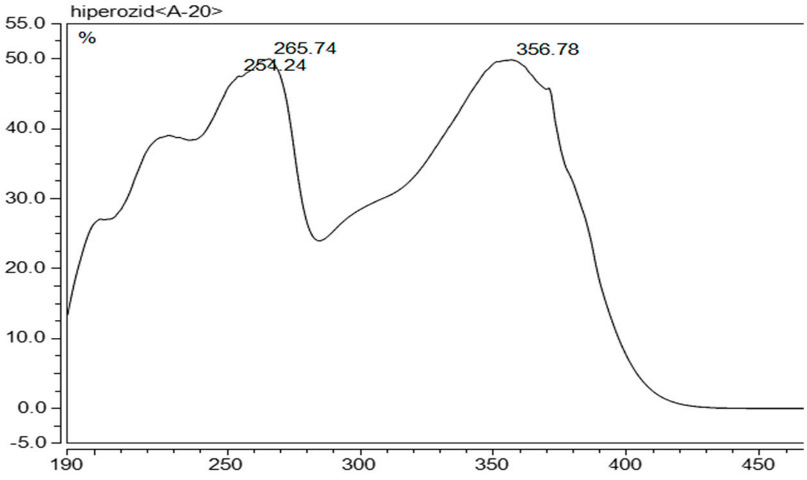

Supplementary Figure S5. UV spectrum for the Hyperoside used standard

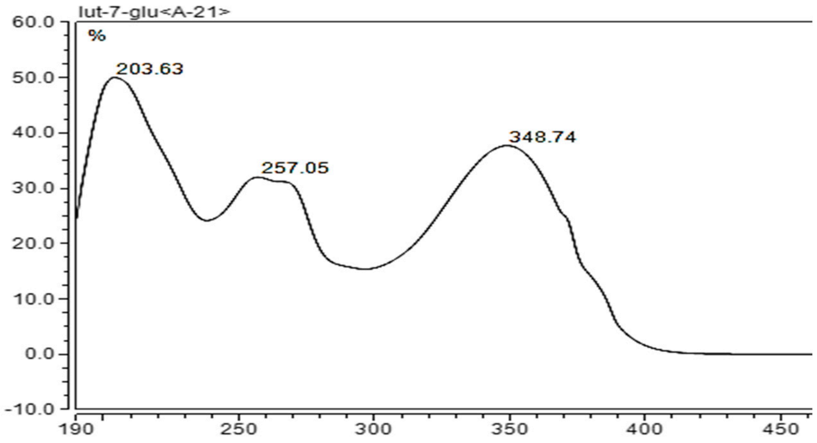

Supplementary Figure S6. UV spectrum for the Luteolin-7-O-glucoside used standard

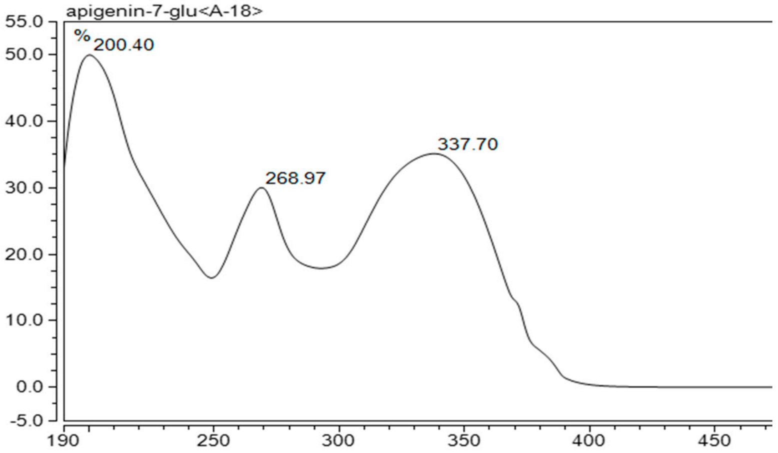

Supplementary Figure S7. UV spectrum for the Apigenin-7-O-glucoside used standard

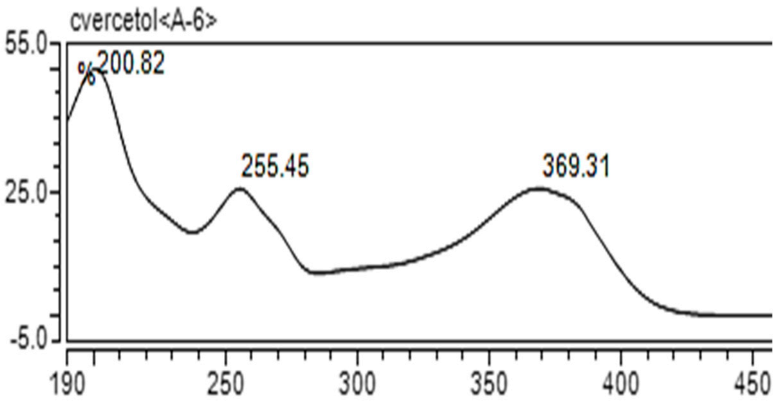

Supplementary Figure S8. UV spectrum for the Quercetin used standard

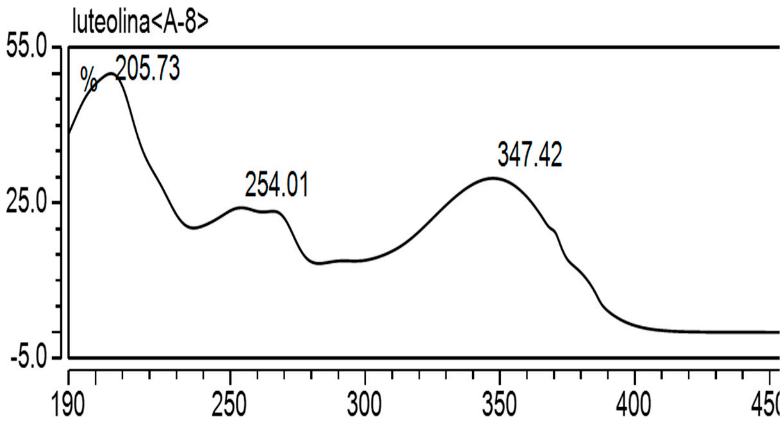

Supplementary Figure S9. UV spectrum for the Luteolin used standard

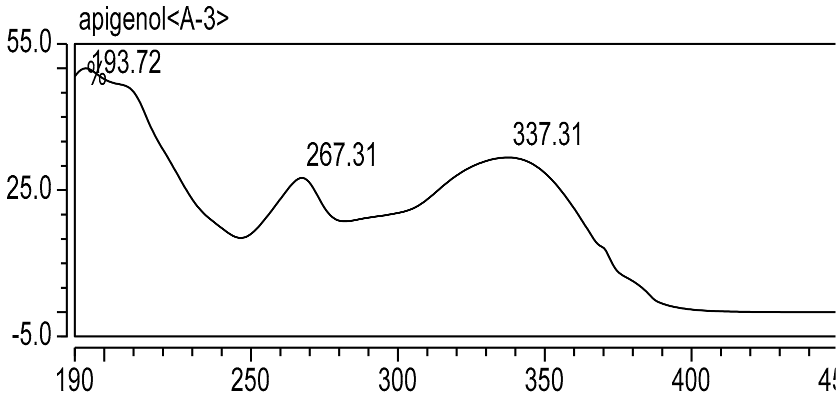

Supplementary Figure S10. UV spectrum for the Apigenin used standard
